# Effects of Low Temperatures on Flexural Strength of Macro-Synthetic Fiber Reinforced Concrete: Experimental and Numerical Investigation

**DOI:** 10.3390/ma15031153

**Published:** 2022-02-02

**Authors:** Stanislav Aidarov, Alejandro Nogales, Igor Reynvart, Nikola Tošić, Albert de la Fuente

**Affiliations:** 1 Smart Engineering Ltd., UPC Spin-Off, Jordi Girona 1-3, 08034 Barcelona, Spain; alejandro.nogales@upc.edu (A.N.); igor.reynvart@estudiant.upc.edu (I.R.); 2 Civil and Environmental Engineering Department, Universitat Politècnica de Catalunya (UPC), Jordi Girona 1-3, 08034 Barcelona, Spain; nikola.tosic@upc.edu (N.T.); albert.de.la.fuente@upc.edu (A.d.l.F.)

**Keywords:** polymeric fiber reinforced concrete, experimental program, temperature variation, residual tensile strength, one-way element, beam, non-linear analysis, modeling

## Abstract

Fiber-reinforced concrete (FRC) is an attractive alternative to traditional steel bar-reinforced concrete structures, as evidenced by the constantly increasing market consumption of structural fibers for this purpose. In spite of significant research dedicated to FRC, less attention has been given to the effects of low temperatures on the mechanical properties of FRC, which can be critical for a variety of structural typologies and regions. With this in mind, an experimental program was carried out to assess the flexural behavior of macro-synthetic fiber-reinforced concrete (MSFRC) at different temperatures (from 20 °C to −30 °C) by means of three-point bending notched beam tests. The tested MSFRCs were produced by varying the content of polypropylene fibers (4 and 8 kg/m^3^). The results proved that the flexural strength capacity of all MSFRCs improved with decreasing temperature. Finite element analyses were then used to calibrate constitutive models following *fib* Model Code 2010 guidelines and to formulate empirical adjustments for taking into account the effects of low temperatures. The outcomes of this research are the basis for future experimental and numerical efforts meant to improve the design of MSFRCs subjected to low temperatures during service conditions.

## 1. Introduction

Fiber-reinforced concrete (FRC) is one of several new types of innovative concretes that can be used for structural purposes in accordance with a number of national and international codes, guidelines, and design recommendations [1,2,3,4,5,6]. The incorporation of fibers in cement-based composites allows the partial or even total substitution of traditional reinforcement (reinforcing steel bars) with a positive effect on the fracture energy of the matrix [7], cracking control [8,9,10,11,12], fire resistance [13,14,15], fatigue [16,17], redistribution capacity [18,19,20,21], and impact resistance [22,23,24,25]. As a result, the application of FRC is already observed in a multitude of structural elements, such as precast tunnel segments [26,27,28], elevated flat slabs [29,30,31], reinforced earth-retaining walls [32], and ground-supported flat slabs for industrial applications [33,34].

**Figure 1:**
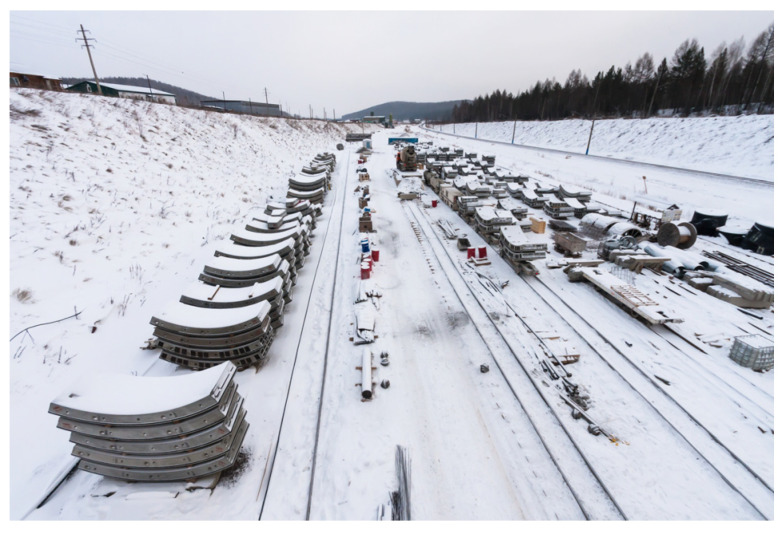
Precast concrete elements subjected to low temperatures (reproduced with permission from [52]).

Moreover, numerous research programs are focused on the material characterization of FRC [35,36,37,38,39] and the further elaboration of both analytical [40,41,42,43,44,45,46] and numerical [47,48,49,50,51] design approaches to suitably evaluate the response of FRC elements under diverse load/boundary conditions. However, the majority of research studies tend to evaluate the mechanical performance of FRC subjected to normal temperature conditions or high/extremely high (e.g., fire) temperatures. In contrast, the behavior of FRC at low temperatures is scarcely analyzed, although there are a number of possible scenarios in which it would be essential to adequately predict its structural response under relatively adverse conditions, e.g., (1) the storage (Figure 1), handling, and transportation of precast tunnel segments in cold regions or (2) the service life of industrial floors for cold-storage warehouses.

Drawing an analogy with plain or traditionally reinforced concrete under these conditions, an increase in compressive and tensile strength can be expected [53,54] along with the embrittlement of a concrete matrix [55]. Taking into account the increased tensile strength of the material, sufficient ductility to the structural element must be provided by the reinforcement once cracking occurs. In the case of FRC, residual tensile strength is mainly responsible for the post-cracking behavior of the material—this property should also be improved as the matrix–fiber interaction enhances with decreasing temperatures as long as the mechanical properties of the fibers are not negatively affected by external conditions.

However, to the authors’ best knowledge, only a few experimental investigations were dedicated to examining the influence of low temperatures on the post-cracking response of FRC with moderate values of compressive strength (up to 60 MPa). Pigeon and Cantin [56] highlighted the slight and significant increase in steel fiber reinforced concrete (SFRC) toughness at −10 °C and −30 °C, respectively. Caballero-Jorna et al. [57] emphasized a minor enhancement of the post-cracking flexural strength of SFRC and MSFRC at −15 °C, whereas Richardson and Ovington [58], on the contrary, stressed a considerably greater flexural strength of both SFRC and MSFRC at −20 °C. Despite the obtained results, there is still a lack of information for modeling possible “temperature–post-cracking flexural strength” relationships for different types of FRC and, more importantly, the adjusted designed procedures to suitably predict the structural response of FRC at low temperatures have still not been analyzed.

With this in mind, the presented research study was conducted, this being comprised of two main parts. Primarily, an experimental program was carried out in order to characterize pre- and post-cracking flexural behavior of FRC at different temperatures (from 20 °C to −30 °C): the standardized three-point bending test (3PBT) on a notched beam (150 × 150 × 600 mm^3^) according to EN 14651 [59]. For the sake of more detailed analysis, two types of FRC were characterized: MSFRC with fiber contents of 4 kg/m^3^ (MSFRC-4) and 8 kg/m^3^ (MSFRC-8).

Thereafter, multi-linear constitutive models were derived pursuant to the *fib* Model Code 2010 [1] in order to simulate experimentally obtained “load–displacement” curves at 20 °C, 0 °C, −10 °C, and −30 °C. Moreover, correction factors to the elaborated constitutive diagrams were identified with the aim of fitting the numerical prediction to the real behavior of the tested beams.

As a result, the experimental outcome enlarged existing databases on FRC mechanical performance and allows practitioners to consider the mechanical properties of FRC when designing for cold climates or conditions where low temperatures are expected. Additionally, the numerical part of the study evidenced the necessity of the correction factors to precisely predict the real behavior of FRC elements reinforced by a given amount/type of fibers.

## 2. Experimental Program

### 2.1. Concrete Mix

The adopted mix (Table 1) corresponded to the normal strength concrete and S3 consistency class, i.e., the measured slump was between 100 and 150 mm [60]. A Portland cement type CEM II-A/L was used for producing the MSFRC mixes. The concrete matrix consisted of three aggregate sizes: sand 0/4, gravel 4/10, and gravel 10/20 from crushed calcareous stone. A lignosulphonate-based plasticizer and a polycarboxylate-based superplasticizer were also added during the material elaboration in order to provide the required workability of the mix.

Based on the described concrete composition, two types of FRC were produced: polypropylene fiber-reinforced concrete with a fiber content of (1) 4 kg/m^3^ (volume fraction 0.425%) and (2) 8 kg/m^3^ (volume fraction 0.850%); Table 2 gathers the essential properties of the used polypropylene fiber (PPF). MSFRC-4 was oriented to industrial floors in which only the minimum reinforcement is required to control cracking due to thermal–hydrometric (temperature and shrinkage gradients) phenomena. In turn, MSFRC-8 was selected to reproduce medium-heavy duty pavements, which, apart from the previously mentioned indirect loads, could be subjected to external loads of notable magnitude.

### 2.2. Test Setup and Testing Procedure

**Figure 2:**
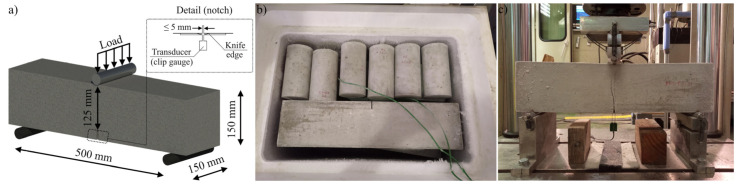
(**a**) 3PBT setup; (**b**) freezing procedure and temperature monitoring; (**c**) 3PBT at −30 °C.

The structural response of the elaborated MSFRC mixes in terms of flexural pre- and post-cracking strengths was analyzed at 20 °C (reference temperature), 0 °C, −10 °C, and −30 °C. This mechanical property of the studied MSFRC was assessed in compliance with EN 14651 [59] (Figure 2a) by testing six notched prismatic beams (150 × 150 × 600 mm) for each temperature magnitude, resulting in 48 tested samples. The casting and demolding (in 24 h) of the prismatic beams in question were followed by the curing of these specimens in a temperature (20 °C) and humidity (95%) controlled chamber for 28 days.

Thereafter, three-quarters of all beams were placed in a laboratory freezer, and each quarter was cooled to 0 °C, −10 °C, and −30 °C, respectively. Importantly, the prismatic samples were equipped with a thermocouple in order to guarantee the target test temperature and, additionally, to monitor its evolution (Figure 2b). Once the required temperature was reached, the specimens were placed in an INSTRON 8505 testing machine (Figure 2c) equipped with a load cell of 100 kN in order to estimate the flexural behavior. The parameters of major concern within the testing procedure were the limits of proportionality (*f_LOP_*), *f_R1_*, and *f_R3_*; *f_LOP_* represented the pre-cracking flexural behavior, whereas the residual tensile strengths *f_R1_* and *f_R3_* were used to derive the constitutive models of the MSFRC for design purposes, being related with serviceability and ultimate limit states, respectively.

## 3. Experimental Results and Discussion

**Figure 3:**
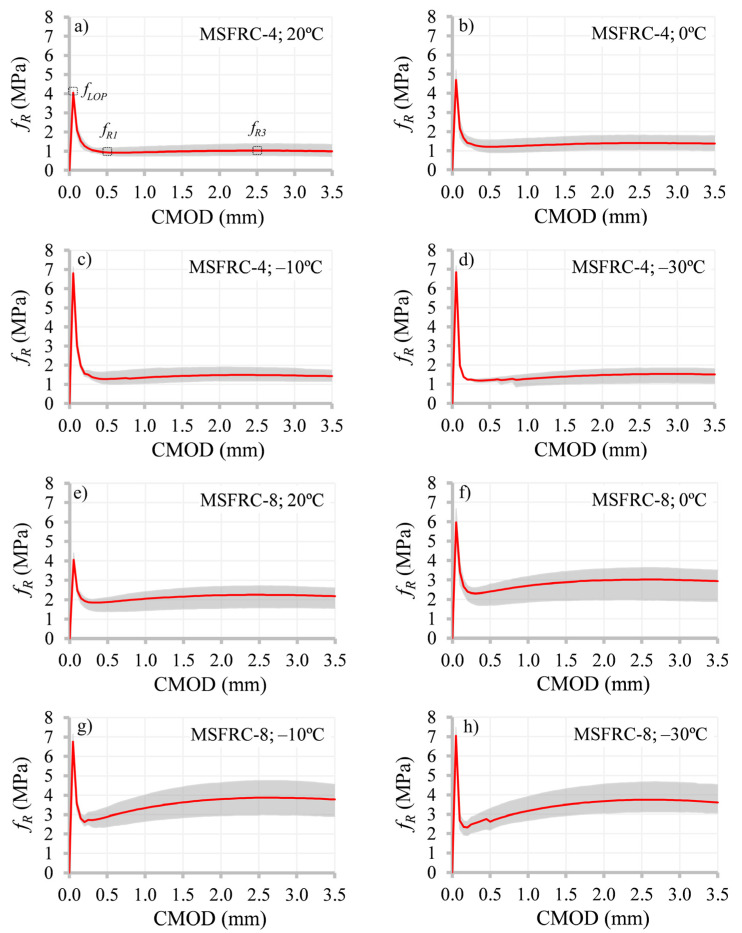
Flexural pre- and post-cracking strength at different temperatures of: (**a**–**d**) MSFRC-4; (**e**–**h**) MSFRC-8.

Figure 3 gathers responses obtained by means of the 3PBT in terms of pre- and post-cracking strength and crack mouth opening displacement (CMOD); average values are highlighted by red lines (based on six tested specimens for each case), whereas result scatter (envelope) is represented by a shaded area. Primarily, pre-cracking behavior was assessed for both MSFRC-4 and MSFRC-8. Taking into consideration that this mechanical property is mainly dependent on the material matrix [61,62] (amount of cement paste and granular skeleton), similar results were expected regardless of the fiber content. 

**Figure 4:**
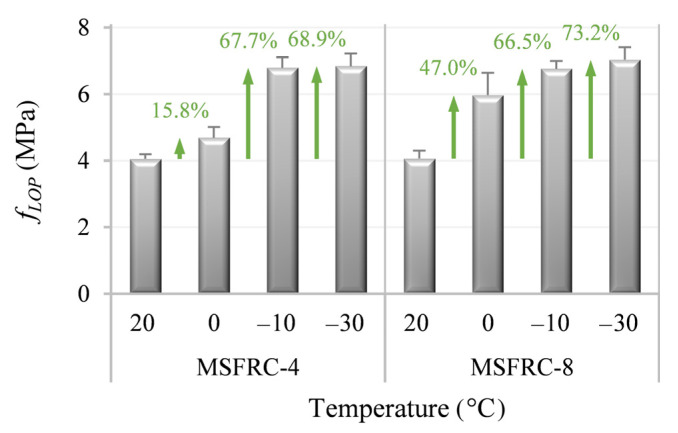
Mean values of *f_LOP_* with corresponding standard deviations of the studied MSFRCs at different temperatures.

This expectation was proven accurate, as seen in Figure 4—the limit of the proportionality of MSFRC-4 and MSFRC-8 at the reference temperature was almost identical. Decreasing the temperature to the threshold value for water to start freezing (0 °C), *f_LOP_* started increasing by 15.8% and 47.0% for MSFRC-4 and MSFRC-8, respectively. Further reduction in the temperature led to a significant enhancement of *f_LOP_*: a total increment of 67.7% and 66.5% was detected for the above listed materials at –10 °C, whereas the temperature magnitude of –30 °C entailed an increment of 68.5% and 73.2% for MSFRC-4 and MSFRC-8, respectively (comparing with *f_LOP_* at 20 °C).

Thereafter, the effect of the temperature variation on the residual tensile strengths (*f_R1_*, *f_R3_*) was estimated. This mechanical parameter, apart from the characteristics of the concrete matrix, depends on a certain number of factors, such as the mechanical properties of the implemented fibers, fiber geometry (having a main effect on anchorage and bond capacity), and fiber distribution and orientation within the critical section. Therefore, the analysis of post-cracking behavior of FRC at low temperatures is a challenging aspect to be investigated.

**Figure 5:**
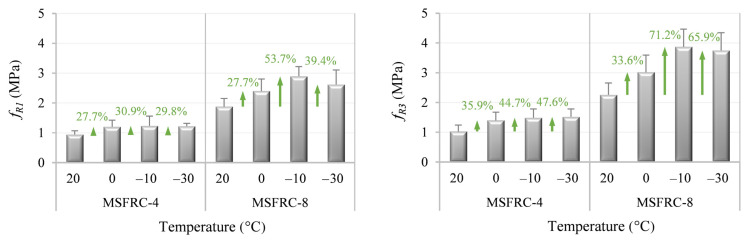
Mean values of *f_R1_* and *f_R3_* with corresponding standard deviations of the studied MSFRCs at different temperatures.

The results that can serve as a base for further investigations are presented in Figure 5. Analyzing the observed structural response of the tested FRC beams, the positive effect of low temperatures on the energy required to produce fiber pull-out can be emphasized, i.e., the concrete matrix that embeds the fibers shrinks with the decrease in temperature, provoking an increase in confinement pressure along the fibers and thus enhancing the anchorage capacity of fibers. This phenomenon, in turn, improves the post-cracking flexural behavior as it was depicted in [63,64]. Importantly, the studied range of temperatures had no detectable negative influence on the mechanical properties of the implemented fibers and, thereby, on the overall performance of the studied FRC beams. 

Additionally, the effect of the higher fiber content on the enhancement rate of the residual tensile strength (due to temperature reduction) can be stressed: the relatively moderate values of residual tensile strengths in the case of MSFRC-4 did not ensure the continuous increment of post-cracking strength, i.e., both *f_R1_* and *f_R3_* presented similar enhancements of this parameter in the range between 0 °C and −30 °C. Contrarily, MSFRC-8 evidenced a significant increment of residual tensile strengths once temperatures surpassed the threshold temperature magnitude of 0 °C—up to 71.2% in comparison with the reference values (at 20 °C). Moreover, the greater increase in *f_R3_* should be highlighted, opposing the enhancement rate due to low temperatures to the increase in *f_R1_*—this phenomenon results from the improvement of the bond capacity in the matrix–fiber interaction that is generally a governing failure mechanism (fiber debonding).

Although the obtained experimental outcome clearly evidenced the increase in pre- and post-cracking flexural strength of the given FRCs, further studies are required to extend the database related to the effect of low temperatures on flexural capacity of the material in question, varying temperature magnitudes, concrete mixes, and fiber type/contents. This will allow to propose a relationship between temperature variation and the flexural strength of FRCs; this relationship, in turn, will allow to characterize the materials at ambient conditions with a subsequent estimation of their potential behavior at more severe conditions.

## 4. Numerical Analysis

In previous sections, experimental tests have proven the positive effect of low temperatures on FRC by increasing post-cracking strength. Based on the authors’ experience, the FRC constitutive equation proposed in the *fib* Model Code 2010 [1], which was set as a reference for design engineers and practitioners to take into account the post-cracking behavior of FRC, needs to be adjusted in order to properly reproduce the latter. The adjustments usually adopted in MSFRC are to reduce residual strength at early stages (for CMOD < 0.5 mm) since the constitutive equation tends to overestimate the flexural post-cracking capacity after cracking, whereas the behavior at larger crack openings tends to be underestimated, and the residual strength needs to be increased [65,66].

**Figure 6:**
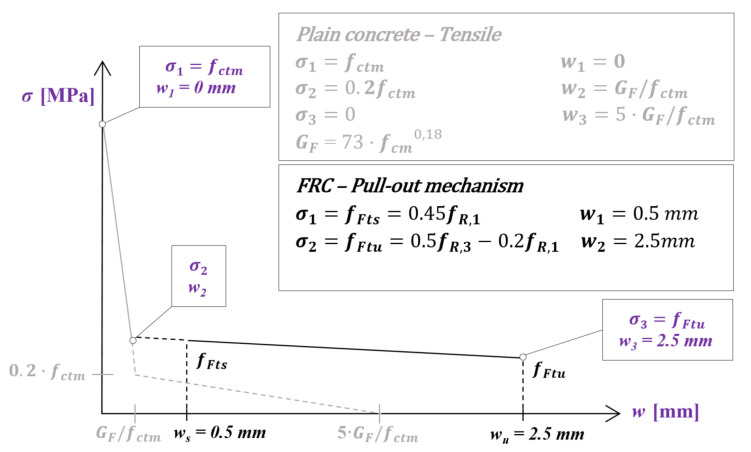
*fib* MC-2010 [1] FRC tensile constitutive equation.

With that in mind, this section presents a non-linear finite element (FE) simulation to obtain and assess the ratio of residual strength (obtained by means of a numerical simulation) to experimental residual strength (*f_R,NL_*/*f_R,EXP_*) for the FRC mixes (MSFRC-4 and MSFRC-8) tested at different temperatures. In order to derive these *f_R,NL_*/*f_R,EXP_* ratios, the strategy adopted is the following: first, non-linear simulations of a beam flexural post-cracking strength test were carried out implementing an FRC-constitutive relationship according to *fib* Model Code 2010 [1]. Figure 6 depicts the schematic representation of the stress-crack width curve for FRC according to the *fib* Model Code 2010 [1]. A full curve is obtained as the combination of the post-cracking response of plain concrete (where *f_ctm_* and *G_F_* stand for mean tensile concrete strength and fracture energy, respectively) and the fiber contribution through the pull-out mechanism, the first point being *σ*_1_ = *f_ctm_* and *w*_1_ = 0 mm, the second point σ_2_ and *w*_2_ (the intersection between the two curves), and the third one *σ*_3_ = *f_ctm_* and *w*_3_ = 2.5 mm. Moreover, since the results did not fit the experimental ones well, the *f_R1_* and *f_R3_* coefficients (to derive constitutive curves according to the *fib* Model Code 2010 constitutive equations) were modified so that the resulting curves fit the values at CMOD of 0.5 and 2.5 mm, which are the crack openings for serviceability and ultimate limit states, respectively. The latter task was undertaken by implementing a back analysis by an iterative trial and error process.

In order to carry out the numerical analysis, non-linear simulations were implemented by means of the commercial finite element (FE) software ABAQUS CAE 2016 [67], as its adequacy in properly reproducing the post-cracking flexural performance of FRC has been successfully proven through an available Concrete Damaged Plasticity (CDP) numerical model [20,66]. CDP is a smeared crack plasticity-based numerical model, which assumes that the main two failure mechanisms are tensile cracking and concrete crushing. Input data are required in terms of uniaxial stress-strain (*σ–ε*) curves for both tensile and compressive behavior. In this study, in order to minimize mesh dependence due to different mesh size, the stress-crack width (*σ–w*) tensile curve was used instead of *σ–ε* [67]. The compressive constitutive curve adopted was proposed in the *fib* Model Code 2010 [1], and the CDP magnitude of the parameters adopted for all the simulations were those proposed in ABAQUS Users’ Manual [67] for plain concrete, which can be found elsewhere [68].

**Figure 7:**
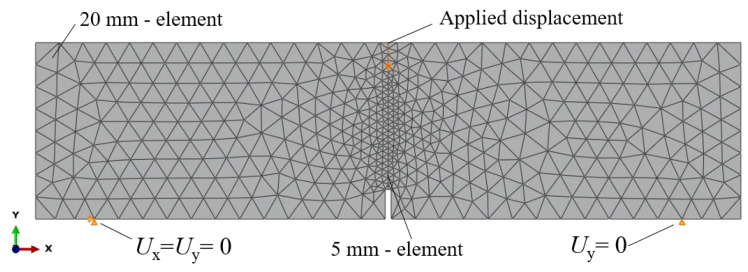
2D FEM model adopted: mesh and boundary conditions considered.

The adopted 2D model considering plain strain conditions is depicted in Figure 7, which shows the loading and boundary conditions along with the mesh. In agreement with the experimental test, the boundary conditions were imposed so that vertical displacement was restrained (*U_y_* = 0) at both supports and horizontal displacement (*U_x_* = 0) in one of them. The loading was applied by means of displacement control using an explicit dynamic algorithm (quasi-static analysis) in order to properly capture the post-cracking performance of FRC. The mesh comprised 485 nodes and 886 triangular linear elements (CPE3) with a mesh size of 20 mm, refined in the mid-section with 5 mm size elements, wherein the mesh size was determined after carrying out a mesh sensitivity analysis.

**Figure 8:**
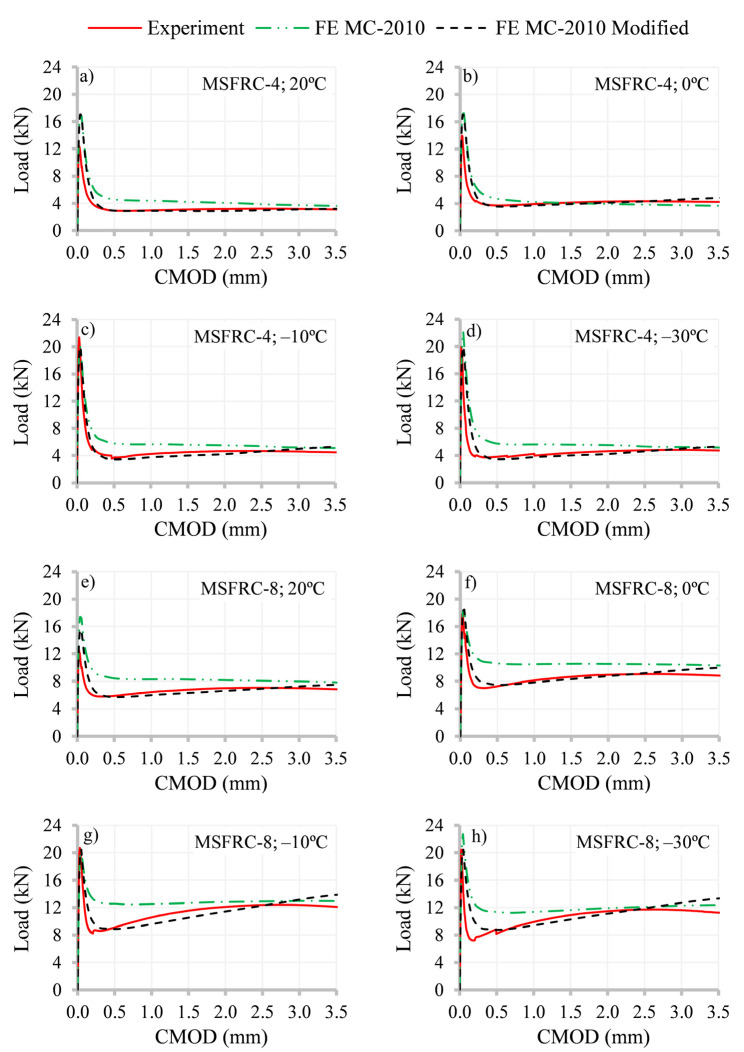
Results for MSFRC-4 and MSFRC-8 at studied temperatures in terms of “load–CMOD”.

The results of the non-linear simulations for MSFRC-4 and MSFRC-8 for all temperatures are plotted in Figure 8. The load–CMOD graphs include three curves: experimental results and two from non-linear simulations (1) implementing the *fib* Model Code [1] constitutive equation (FE MC-2010, derived using *f_R1_* and *f_R3_* obtained from the experimental tests presented in Section 3) and (2) using the constitutive curve (FE MC-2010 Modified), adjusted so that the results fit the experimental curve at 0.5 and 2.5 mm. As can be seen, the simulations with FE MC-2010 Modified only fit the experimental data at these points, and hardening is produced in a linear way. Based on the authors’ experience, in those cases where the hardening is produced in a curved way, more points would be necessary in the constitutive equation in order to better adjust the experimental outcome [65,66].

It is worth noticing that the ultimate displacement in the *fib* Model Code [1] constitutive curve is 2.5 mm (set as the stress for ultimate limit state analysis). However, in this research study, after 2.5 mm, the constitutive curve smoothly decreases to zero stress, set at *w* = 5 mm, in order to better capture the flexural bearing capacity of MSFRC at latter stages. Without the last branch of the curve, the maximum post-cracking load of the tests, after the drop due to cracking, cannot be captured since it is produced for crack openings higher than 2.5 mm [65,66].

Table 3 gathers more detailed information regarding *f_R1_* and *f_R3_* parameters for MSFRC-4 and MSFRC-8, respectively. These parameters were used for deriving the constitutive curves obtained from the experimental data and by means of the back analysis. In addition, the table also presents the *f_R,NL_*/*f_R,EXP_* ratios for each MSFRC mix and temperature.

Based on the outcome presented in the table, it can clearly be seen that the ranges of the *f_R,NL_*/*f_R,EXP_* ratios for either *f_R1_* or *f_R3_* are quite narrow, particularly for *f_R1_*. In this sense, it could be stated that the *f_R,NL_*/*f_R,EXP_* ratios are constant despite the increasing post-cracking strength with decreasing temperature. In view of this, in cases where no data are available for low temperatures, and the structure is expected to be subjected to large temperature variations, the same *f_R,NL_*/*f_R,EXP_* ratio (for either *f_R1_* and *f_R3_*) at reference temperature could be taken for the MSFRC design.

**Figure 9:**
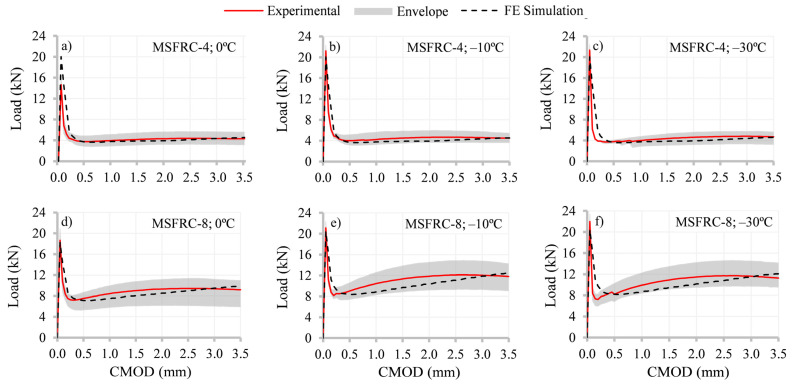
Results in terms of load–CMOD for MSFRC-4 and MSFRC-8 using *f_R,NL_*/*f_R,EXP_* at reference temperature to derive the constitutive curves.

In order to verify this assumption, in Figure 9 were plotted the FE simulations using the residual strengths from the tests at each temperature and multiplied by the *f_R,NL_*/*f_R,EXP_* ratio (for either *f_R1_* and *f_R3_*) at 20 °C (i.e., *f_R1,NL_*/*f_R1,EXP_* = 0.65 and 0.75 for MSFRC-4 and MSFRC-8, respectively, and *f_R3,NL_*/*f_R3,EXP_* = 1.10 for both solutions). As can be seen, the differences at CMOD 0.5 and 2.5 mm have a deviation lower than 10% in all cases (Figure 9) which is assumed to be acceptable for engineering design. Moreover, taking into consideration the scatter in the experimental tests of MSFRC post-cracking performance, these new simulations are inside the envelope, which means that this approach is representative of the mechanical performance of each solution of MSFRC.

It is worth noticing that, in a hypothetical situation in which no tests were performed at low temperatures, *f_R1_* and *f_R3_* at different temperatures could be obtained based on the established relationship “temperature variation–flexural strength” that is to be elaborated once the broader database of the experimental results is developed, as proposed in Section 3.

## 5. Conclusions

In this paper, an experimental program was described following an analysis of low temperature effects on the pre- and post-cracking flexural behavior of macro-synthetic fiber reinforced concrete (MSFRC). In total, 48 prismatic notched beams were tested under a three-point bending configuration, varying temperatures from 20 °C to −30 °C. Moreover, numerical analyses were performed to verify the suitability of the current constitutive models suggested by the *fib* Model Code 2010 for simulating the pre- and post-cracking response of the MSFRCs tested at low temperatures. The following conclusions may be derived from the obtained results:

- Low temperatures led to an increase in the required energy to produce fiber pull-out, and therefore, post-cracking flexural behavior was enhanced: *f_R1_* and *f_R3_* increased to 54% and 71%, respectively, for temperatures below 0 °C.
- A greater increase in *f_R3_* at low temperatures (in comparison with the observed values of *f_R1_*) was observed. This could be due to the confinement effect caused by the shrinkage of the matrix embedding the fibers. This effect seemed to lead to a higher fiber bond (higher matrix–fiber friction). This outcome is of paramount importance for design procedures at ultimate conditions.
- The numerical analysis adopting the multi-linear constitutive model that is suggested by the *fib* Model Code 2010 evidenced a certain overestimation of the real flexural behavior in cases of studied MSFRC prismatic beams. This phenomenon led to the requirement of introducing correction factors to properly simulate the structural response of the elements in question. Importantly, the imposed correction factors (for both *f_R1_* and *f_R3_*) were almost identical despite the temperature variation, although the fiber content did have an effect on these values.

The outcome of the described research program reveals the enhanced performance of the given FRCs in terms of pre- and post-cracking flexural strengths—therefore, these phenomena should be taken into account during the design procedures of elements that are to be subjected to low temperatures during transient or in-service conditions. However, further investigation is required to expand the experimental database related to the behavior of FRCs at low temperatures. This will allow to evaluate the “temperature–pre- and post- flexural strength” relationships so that designers and practitioners will only need to carry out the characterization of the required material at ambient conditions (20 °C) in order to adequately predict structural behavior at low temperatures.

## Figures and Tables

**Table 1:** Composition of studied MSFRC.

Materials	MSFRC-4	MSFRC-8
CEM II-A/L 42.5R (kg/m^3^)	310	310
Coarse aggregate 10/20 (kg/m^3^)	690	680
Coarse aggregate 4/10 (kg/m^3^)	127	125
Fine aggregate 0/4 (kg/m^3^)	1025	1025
Water-cement ratio	0.58	0.58
Additives (% on cement content)	1.2	1.5
Synthetic fibers (kg/m^3^)	4	8

**Table 2:** Properties of the implemented fiber.

Property	PPF	Representation
Material	Transparent polypropylene	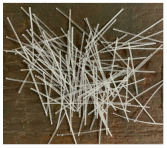
Shape	Embossed monofilament	
Diameter (mm)	0.85	
Length (mm)	48	
Aspect ratio	56.5	
Number of fibers per kg	41,200	
Tensile strength (MPa)	>400	

**Table 3:** *f_RNL_*/*f_REXP_* ratio for MSFRC-4 and MSFRC-8 at studied temperatures.

	Temperature (°C)	MSFRC-4			MSFRC-8		
		*f_R,EXP_* (MPa)	*f_R,NL_* (Mpa)	*f_R,NL_*/*f_R,EXP_*	*f_R,EXP_* (Mpa)	*f_R,NL_* (Mpa)	*f_R,NL_*/*f_R,EXP_*
*f_R1_*	20	0.94	0.60	0.64	1.88	1.35	0.72
	0	1.20	0.80	0.67	2.4	1.80	0.75
	−10	1.23	0.80	0.65	2.89	2.25	0.78
	−30	1.22	0.80	0.66	2.83	2.25	0.80
*f_R3_*	20	1.03	1.03	1.00	2.26	2.40	1.06
	0	1.40	1.60	1.14	3.02	3.20	1.06
	−10	1.49	1.80	1.21	3.87	4.60	1.19
	−30	1.52	1.80	1.18	3.75	4.60	1.23

## Data Availability

The data presented in this study are available on request from the corresponding author.
